# Controlled levels of protein modification through a chromatography-mediated bioconjugation†
†Electronic supplementary information (ESI) available: Full experimental procedures and additional characterization data. See DOI: 10.1039/c4sc03790a



**DOI:** 10.1039/c4sc03790a

**Published:** 2015-02-27

**Authors:** Richard L. Kwant, Jake Jaffe, Peter J. Palmere, Matthew B. Francis

**Affiliations:** a Department of Chemistry , University of California at Berkeley , Berkeley , California 94720-1460 , USA . Email: mbfrancis@berkeley.edu; b Materials Sciences Division , Lawrence Berkeley National Laboratory , Berkeley , California 94720 , USA

## Abstract

Synthetically modified proteins are increasingly finding applications as well-defined scaffolds for materials. In practice it remains difficult to construct bioconjugates with precise levels of modification because of the limited number of repeated functional groups on proteins. This article describes a method to control the level of protein modification in cases where there exist multiple potential modification sites. A protein is first tagged with a handle using any of a variety of modification chemistries. This handle is used to isolate proteins with a particular number of modifications *via* affinity chromatography, and then the handle is elaborated with a desired moiety using an oxidative coupling reaction. This method results in a sample of protein with a well-defined number of modifications, and we find it particularly applicable to systems like protein homomultimers in which there is no way to discern between chemically identical subunits. We demonstrate the use of this method in the construction of a protein-templated light-harvesting mimic, a type of system which has historically been difficult to make in a well-defined manner.

## Abstract 2

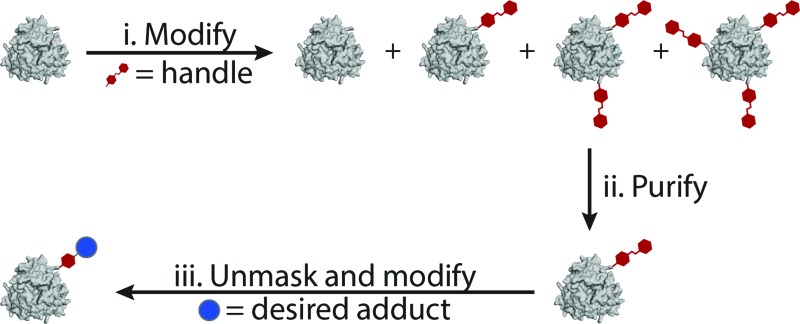
This article introduces a method to control levels of protein modification through a chromatography-mediated bioconjugation.

## Introduction

Protein bioconjugates continue to grow in prevalence and importance, and recently they have found use as therapeutics,^1^ chemical sensors,^2^ scaffolds for new materials,^3^ and tools for basic research.^4^ The increasing complexity of these materials and their applications is predicated on the development of selective and quantitative methods for protein modification. For example, it is known that the drug loading of antibody–drug conjugates affects their efficacy.^5,6^ However, because antibodies are large proteins with multiple chains, common methods such as NHS-ester chemistry or maleimide chemistry cannot be used to modify these proteins in a controlled manner. Chemists have addressed this problem by developing chemical methods that target a limited number of potential modification sites. Such methods select for sites that are either rare,^7,8^ unique,^9–13^ introduced,^14–18^ or in close proximity to a directing site.^19^ These techniques have allowed the construction of many well-defined bioconjugates because they have excellent functional group tolerance, can reach high conversion, and are highly selective.

In practice, cases remain in which it is difficult to control protein modification because a single reactive site cannot be identified: a particularly salient example is the modification of protein homomultimers, where chemically identical subunits cannot be differentiated. In these situations one might consider an alternative approach in which a desired bioconjugate is purified from a statistical product mixture. Such a modify-and-purify scheme eases the requirements for high selectivity on the modification chemistry, and therefore allows access to well-defined protein bioconjugates in cases where no selective chemical methods exist. Moreover, this approach complements existing strategies by enabling the removal of minor side products that arise using even the most selective bioconjugation reactions. This approach is seldom used^20–24^ because most existing methods for protein purification—including gel filtration, ion-exchange chromatography, and hydrophobic interaction chromatography—poorly discern the small differences in polarity, charge, and size brought about by modification with an arbitrary small molecule. Only hydrophobic interaction chromatography has been shown in some cases to separate proteins based on their degree of modification,^22–24^ but this technique usually does not result in sufficient separation and is dependent on the properties of the added functional group.^25^

**Fig. 1:**
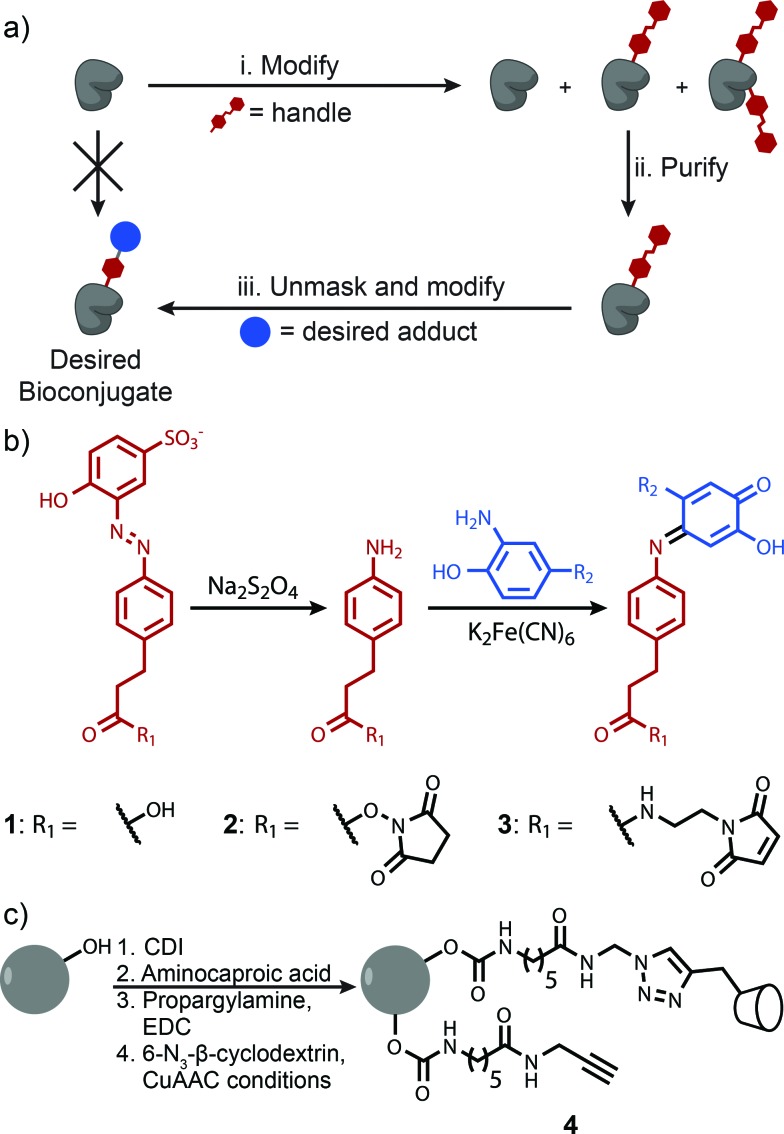
(a) Proposed method for handle-assisted protein modification. The protein is first modified with a handle (i). Proteins with the desired level of modification are isolated based on an affinity interaction with this handle (ii). Innate reactivity in the handle is unmasked, and the handle is modified with a desired bioconjugate (iii). (b) Azo handle. Azo **1** binds β-cyclodextrin with a binding constant sufficient for purification. Its cleavage under mild reducing conditions affords an aniline derivative that can be coupled to many moieties through highly efficient chemistry. (c) Functionalization of 90 μM sepharose CL-4B with β-cyclodextrin.

This difficulty can be overcome by tagging a protein with an affinity handle that also serves as a site for further modification. In this handle-assisted approach, proteins tagged with a desired number of chemical handles are first isolated from a crude reaction mixture using affinity chromatography. After purification, the chemical handles are selectively elaborated to access a sample of well-defined bioconjugate modified with an arbitrary chemical moiety (Fig. 1a). Such a method allows the controlled modification of a protein that has more potential modification sites than the desired number of modifications. In this report, we introduce such a technique and demonstrate its ability to control the modification levels of several monomeric proteins that are modified with NHS-ester chemistry. We then apply this methodology to a particularly challenging bioconjugation target—a synthetic light-harvesting mimic with a precise ratio of dyes templated by a protein homotrimer.

## Results and discussion

The key to the successful implementation of this technique is the handle, for it must participate in a specific affinity interaction and have reactivity that allows further modification. Based on these requirements, we identified azo **1** as a candidate handle (Fig. 1b). This molecule binds to β-cyclodextrin with a stability constant of 10^3.5^ M^–1^, and theoretical calculations indicate that this value is ideal for separations with affinity chromatography (Fig. S1†). Moreover, azo **1** can be cleaved under mild reducing conditions^26^ to afford an aniline derivative. This moiety can participate in an oxidative coupling, a method for the site-selective modification of proteins that commonly reaches high conversion in less than 30 min.^15,16^ This chemistry has excellent functional group tolerance, has been shown to work with a variety of substrates, and has been used successfully in demanding couplings.^3^

β-Cyclodextrin was well-suited to be the binding partner of azo **1** because prior work has shown that it interacts minimally with most proteins.^20,27^ As a result, β-cyclodextrin immobilized on a resin should separate proteins modified with azo **1** derivatives based primarily on their degree of modification. Sepharose CL-4B was identified as an ideal solid support because of its excellent chemical resistance, large pore size, and ability to be modified without the introduction of charged groups that could impede the elution of some proteins through ion-exchange interactions. β-Cyclodextrin was immobilized on this substrate using a copper(i)-catalyzed alkyne–azide cycloaddition^28^ to afford resin **4** (Fig. 1c). Several resins were prepared in which the linker concentration varied from 9 to 32 mM, and in all cases the concentration of immobilized β-cyclodextrin was determined to be 1–2 mM by performing pulldown experiments with an isomer of azo **1** (Fig. S7†). The excess of unreacted alkyne-terminated linker was found to interact favorably with the azo **1** without increasing the retention of unmodified proteins (Fig. S8†). This feature allowed us to increase the separation between unmodified and modified proteins by increasing the linker concentration. A total linker concentration of 15 mM was found to be optimal because it provided sufficient resolution without excessively large retention times (Fig. S8d†). Initial characterization studies also revealed that this resin interacted minimally with unmodified proteins.

**Fig. 2:**
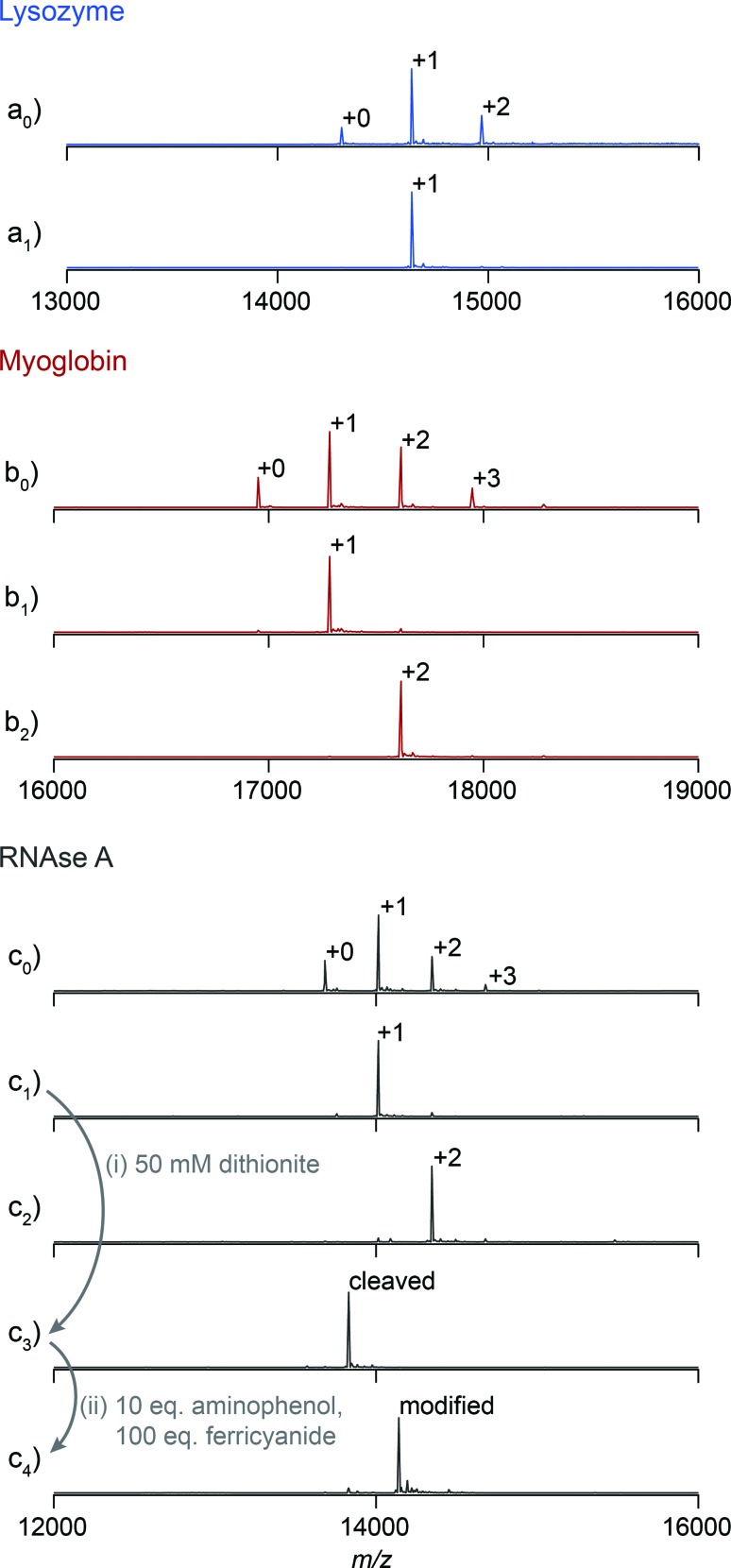
Reconstructed ESI-TOF mass spectra of crude (a_0_, b_0_ and c_0_) and purified bioconjugates tagged with azo NHS-ester **2**. Using handle-assisted purification, it was possible to isolate singly modified samples of each protein (a_1_, b_1_ and c_1_), and doubly modified samples of myoglobin and RNAse A (b_2_ and c_2_). As a demonstration of the potential for the modification of these tagged proteins, the azo handle on singly modified RNAse A was cleaved (i) and coupled to adamantane aminophenol using an oxidative coupling (ii).

We first explored the potential of this technique to control the number of modifications on monomeric proteins. NHS-ester chemistry was selected to perform the initial tagging with azo **1** because it is frequently used, yet it often results in over modification. Lysozyme, myoglobin, and RNAse A were tagged using azo NHS-ester **2**, and LC-MS analysis of the products indicated that all three showed characteristic product mixtures with up to three copies of the azo handle on some proteins (Fig. 2a_0_–c_0_). These samples were then purified by elution from a 25 cm column packed with resin **4** using a linear gradient of 0–10 mM β-cyclodextrin. This procedure resulted in separation of the protein bioconjugates based on their degree of modification with azo NHS-ester **2** (Fig. S9†). Subsequent LC-MS analysis of selected fractions revealed that in all cases it was possible to isolate samples of singly modified protein (Fig. 2a_1_–c_1_), and for myoglobin and RNAse A it was also possible to isolate doubly modified protein (Fig. 2b_2_ and c_2_). The purity of these samples was calculated by comparing the areas of the peaks corresponding to each modification level in Fig. 2, and they ranged from 91% in the case of doubly modified RNAse A to 98% in the case of singly modified lysozyme. As an example of a typical yield for this process, we quantified the amount of singly modified RNAse A that was recovered. The crude sample contained 35% singly modified protein. Purification yielded 72% of the theoretical maximum amount of singly modified protein as determined by UV/vis analysis. Given that some singly modified RNAse A eluted in fractions with unmodified or doubly modified protein, this yield highlights that little protein is lost on the column during purification. The overall yield of singly modified protein was 25% of the total protein.

Previous work in our lab has shown that these isolated samples can be elaborated with an arbitrary moiety to high conversion.^15,16^ As an example, a sample of singly tagged RNAse A was exposed to sodium dithionite to unmask the aniline functionality of the azo handle (Fig. 2i). Cleavage of the azo took place in less than one minute and resulted in complete conversion without the reduction of any of the four disulfide bonds of RNAse A. This sample was then exposed to adamantane *o*-aminophenol under oxidizing conditions to install one adamantyl group on each protein (Fig. 2ii). The reaction reached 94% conversion, and the overall purity of the singly modified conjugate was 89%. Given that the initial purity of RNAse A modified with one azo moiety was about 92%, these results highlight that elaboration of the cleaved azo handle can be accomplished at high enough yield to maintain the purity of isolated samples after the purification step.

**Fig. 3:**
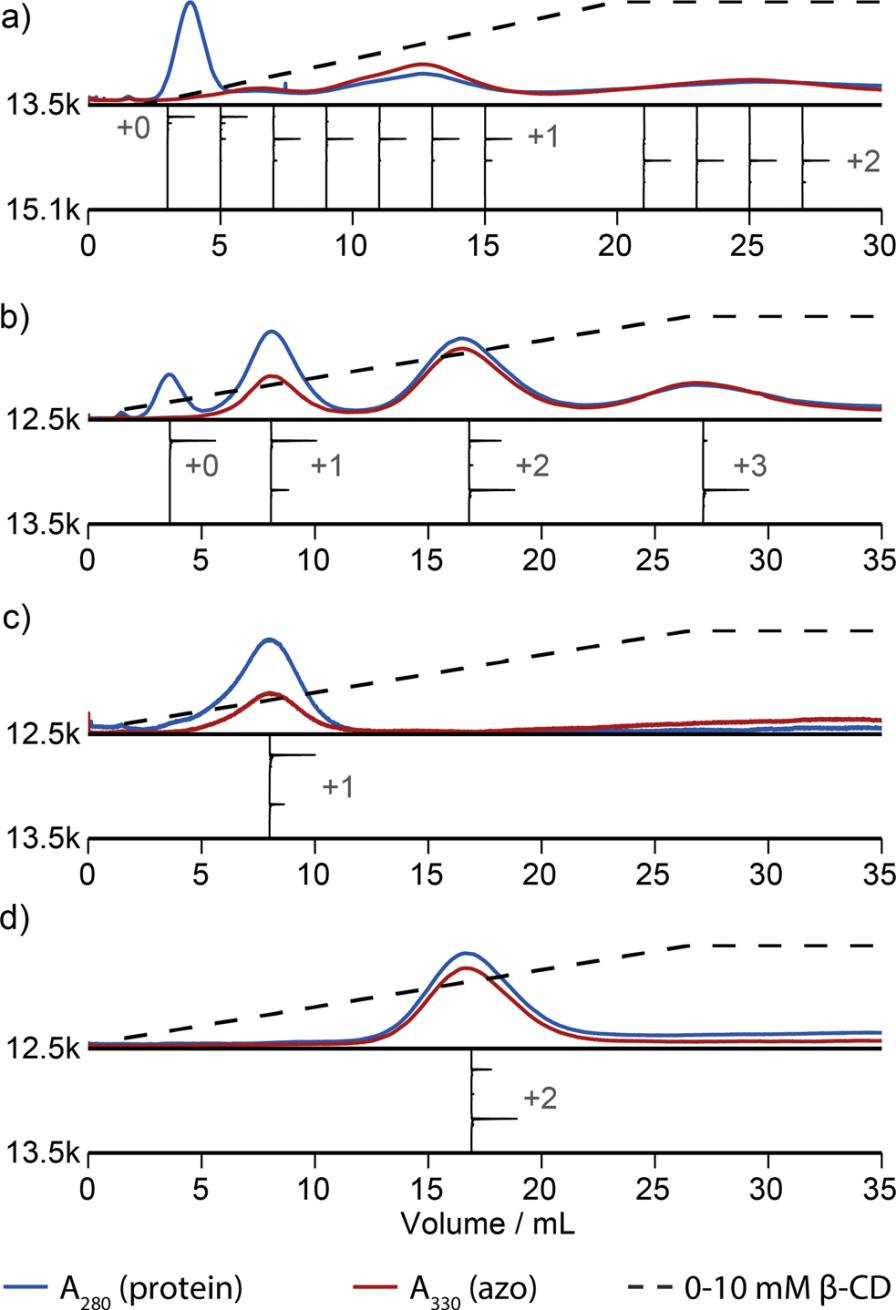
Handle-assisted purification results for (a) RNAse A modified with NHS-ester **2**, (b) the Mth1491 trimer modified with maleimide **3**, (c) re-analysis of the isolated singly-modified Mth1491 species, (d) re-analysis of the isolated doubly-modified Mth1491 species. Below each trace appear reconstructed ESI-TOF mass spectra of selected fractions, rotated 90° clockwise.

Fig. 2 illustrates that this method can be used to isolate proteins with a particular number of modifications. However, this method discerns poorly between proteins that are modified in different locations. For example, inspection of the elution profile of RNAse A reveals that each modification level is resolved, but that singly modified protein elutes as a combination of at least three overlapping peaks between 5 and 15 mL (Fig. 3a). In comparison, purification of N-terminally modified RNAse A—which can be singly modified at only one location—results in only one Gaussian peak corresponding to singly modified protein (Fig. S9†). These results, and similar results from myoglobin, indicate that proteins modified in different locations can have different effective binding constants, likely as a result of secondary interactions between the resin and the local protein environment around the handle. Improvement in the resolution of the affinity step could allow the selection of proteins modified in a particular location or subset of locations, and we are actively exploring alternative resins that could allow this possibility.

Of particular interest to us was the use of this technique to control the modification of protein homomultimers. Our research group has had a longstanding interest in the modification of these proteins because they can be used to mimic natural light-harvesting systems.^4,11^ Such mimics are typically composed of pairs of fluorescent dyes that participate in Förster resonance energy transfer (FRET) and whose arrangement is templated by proteins such as the homotrimer Mth1491, the MS2 viral capsid,^29^ or tobacco mosaic virus coat protein.^4,11^ Because the modification sites on these proteins have identical reactivity, it is impossible to control the arrangement and ratio of dyes on each multimeric assembly at the single molecule level. For example, in the case of the homotrimer Mth1491, an attempt to produce a sample of protein with a 1 : 2 ratio of dyes would result in a statistical mixture of proteins with 0 : 3, 1 : 2, 2 : 1, and 3 : 0 ratios of dyes, with the amount of each species following the binomial distribution. As a result, our studies have been limited to ensemble averages of statistical mixtures of products.

Handle-assisted protein modification could allow controlled modification of multimeric complexes, especially if they are composed of a small number of monomers and therefore have a limited number of potential modification sites. We set out to explore this possibility by using handle-assisted protein modification to create homogeneous samples of Mth1491 with 1 : 2 and 2 : 1 ratios of two FRET pairs. Originally identified in the genome of *Methanobacterium thermoautotrophicum*, Mth1491 was obtained *via* expression in *E. coli*.^30^ To introduce sites for chemical modification, cysteine residues were added to position 92 on each monomer. Optimization studies showed that this site could be modified selectively over the two endogenous cysteines in positions 70 and 72 (Fig. S10†).

A column packed with resin **4** allowed the separation of a product mixture of Mth1491 that was modified to about 50% completion with azo maleimide **3** (Fig. 3b). This chromatogram highlights the difficulties traditionally associated with the modification of these complexes. Each distinct peak corresponds to one of the four distinct modification states of the trimer—unmodified, singly modified, doubly modified, and triply modified—and each species is present in significant abundance. Because the trimer disassembles upon LC-MS analysis, the mass spectrum of each peak shows a ratio of unmodified to singly modified monomers that is consistent with each modification state. For this reason it is also difficult to characterize the homogeneity of these types of samples, and this chromatographic analysis is one of the few methods that can enumerate the modification states of a multimeric protein. To exemplify typical yields during this process, we quantified the amount of doubly modified Mth1491 trimer isolated from one purification. From a 1000 μL sample of 100 μM protein modified to about 50% completion, we isolated 27% of the original protein from the doubly modified peak. To confirm the purity and stability of each isolated sample of Mth1491, these samples were repurified (Fig. 3c and d). The resulting chromatograms illustrate the reliability of this technique in isolating singly and doubly tagged Mth1491 and indicate that the protein stayed folded and assembled during purification, handling, and storage.

**Fig. 4:**
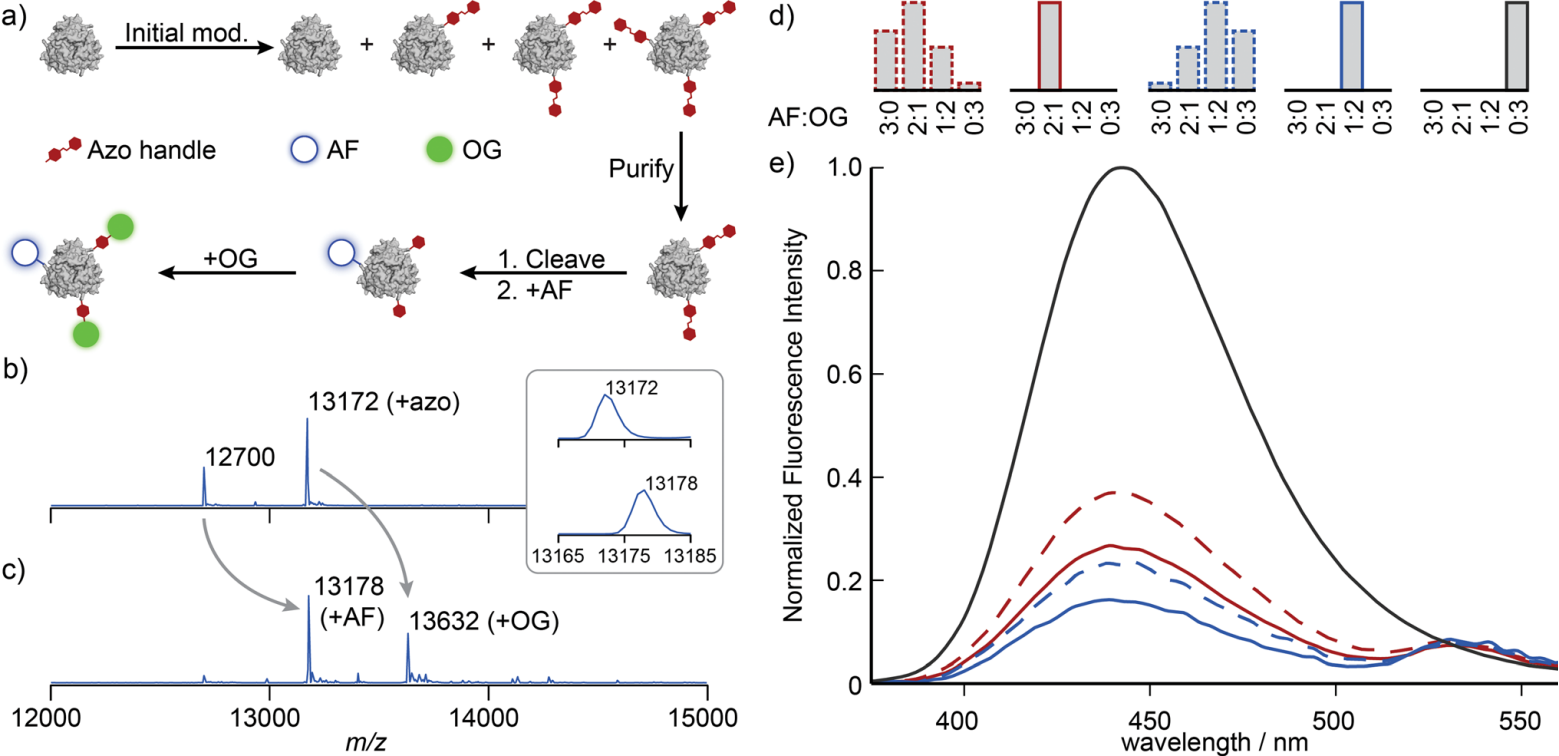
(a) Schematic representation of dual modification of Mth1491 with AF and OG *via* handle-assisted purification and conjugation. (b and c) Reconstructed ESI-TOF mass spectra for the modification of doubly tagged Mth1491 (b) with AF and OG (c). (d) Histograms representing the composition of the samples whose fluorescence properties were measured. The two samples prepared using a handle-assisted strategy consist of a single species (solid borders), whereas the samples prepared using conventional strategies are composed of a statistical mixture. (e) Emission spectra of protein-templated dyes upon excitation at 365 nm. In both cases the homogeneous samples prepared using the handle-assisted strategy exhibit greater quenching, which is characteristic of greater proximity between the two dyes.

With singly and doubly tagged Mth1491 in hand, we continued the handle-assisted modification of these proteins with Alexa Fluor 350 (AF, donor) and Oregon Green 514 (OG, acceptor) as outlined in Fig. 4a. After cleavage of the azo handle, the remaining unmodified cysteines at position 92 were modified with AF maleimide. OG was then coupled to the resulting anilines using an oxidative coupling. We have shown previously that cysteines are modified by oxidized *o*-aminophenols.^16^ In the case of Mth1491, the presence of two endogenous cysteines results in double modification of 18–35% of monomers when 5 to 10 equivalents of *o*-aminophenol are used. To prevent this undesired overlabeling, the protein was protected with Ellman's reagent prior to oxidative coupling, and then subsequently deprotected with TCEP after the reaction. To verify that this protection did not affect the assembly of Mth1491, dynamic light scattering was used to determine that the average particle size increased from 5.5 ± 0.3 nm to 7.4 ± 0.9 nm during the protection step. This increase in diameter is consistent with the modification of the surface of the protein and indicates that the protein was still assembled as a trimer. Fortunately, many proteins will not require this protection step because their cysteines are buried or participate in disulfide bonds.

Illustrative mass spectra for the construction of the 1 : 2 AF : OG sample are shown in Fig. 4b and c. These spectra indicate high conversion for the addition of AF and OG (the peak at 13 172 Da completely disappears, see inset). We presume that changes to the ionizability of the monomers as a result of conjugation to the dyes are responsible for the change in the ratios of the peak heights.‡
‡In our experience these protein–dye conjugates ionize poorly compared to the unmodified and aniline-bearing proteins, with total ion counts that are about an order of magnitude lower. Of the minor peaks found along the baselines, two were identified as the unmodified Mth1491 monomer and the Mth1491 monomer bearing an aniline moiety. However, the abundance of these species was not quantified in light of the poor ionizability of these conjugates. Measurement of the absorbance spectra indicated that AF and OG were present in the expected ratios and supported this interpretation.

The fluorescence properties of these systems were then characterized by considering the quenching of the AF donor. The concentration of AF dye was first calculated from these samples' absorbance spectra after removing the contributions of OG and a minor amount of scatter. Emission spectra were measured upon excitation at 365 nm, and these data were smoothed and corrected for baseline artifacts. These emission spectra were normalized by the concentration of AF dye, and the data were plotted relative to the emission spectrum of a Mth1491 trimer bearing three AF dyes (Fig. 4d and e, solid lines). Both the 2 : 1 an the 1 : 2 AF : OG systems show quenching of the AF donors that is indicative of energy transfer, with efficiencies of 73 and 84%, respectively. Assuming random orientations of the dyes, we computed their Förster radius to be 4.9 ± 0.05 nm. These values for efficiency correspond to distances of 4.1 and 3.7 nm between the dyes. Such lengths are consistent with the fact that the dyes are templated by Mth1491, which has a distance between its cysteines at position 92 of 4.1 nm.

For the purpose of comparison, we constructed systems whose dye content was the same as for the 2 : 1 and 1 : 2 AF : OG samples, but without the purification step shown in Fig. 4a. Obtaining protein initially modified with the correct amount of azo maleimide **3** proved challenging, and we eventually resorted to preparing a number of samples with different modification levels and selecting the correct one. This exercise alone illustrates the difficulties associated with obtaining protein bioconjugates with precise levels of modification, for ∼80% of the protein modified during this process was not used. Construction of the two-dye systems in this way resulted in statistical mixtures of trimers with the four possible combinations of AF and OG that result from traditional strategies for protein modification (Fig. 4d, dashed lines). The emission spectra of these samples indicate reduction in efficiency of energy transfer by 14 and 8%, respectively (Fig. 4d and e, dashed lines). Reduction of FRET efficiency is consistent with the increased separation between the AF donor and the OG acceptor that would result from larger population of the 3 : 0 and 2 : 1 AF : OG systems. These results illustrate the difficulties associated with the controlled modification of homomultimeric proteins and underscore the utility of handle-assisted protein modification in producing well-defined nano-scale materials.

## Conclusions

This article reports a method for the construction of well-defined protein bioconjugates through handle-assisted protein modification. This method relies on the tagging of a protein with a specific affinity handle that allows purification and subsequent modification of the protein. Through this procedure it is possible to control the degree of modification of both monomeric and multimeric proteins, even when the proteins are modified with nonspecific reagents such as NHS-esters. In the case of homomultimeric proteins, this method is to our knowledge the only way to control the number of modifications. We are actively pursuing the development of this method to enable the site-selective modification of proteins, and we anticipate that this method will be of substantial synthetic utility in making protein-based materials of increasing complexity.

## References

[cit1] Filpula D. (2007). Biomol. Eng..

[cit2] Clarke J., Wu H., Jayasinghe L., Patel A., Reid S., Bayley H. (2009). Nat. Nanotechnol..

[cit3] Wu W., Hsiao S. C., Carrico Z. M., Francis M. B. (2009). Angew. Chem., Int. Ed..

[cit4] Ma Y.-Z., Miller R. A., Fleming G. R., Francis M. B. (2008). J. Phys. Chem. B.

[cit5] Hamblett K. J., Senter P. D., Chace D. F., Sun M. M. C., Lenox J., Cerveny C. G., Kissler K. M., Bernhardt S. X., Kopcha A. K., Zabinski R. F., Meyer D. L., Francisco J. A. (2004). Clin. Cancer Res..

[cit6] Junutula J. R., Raab H., Clark S., Bhakta S., Leipold D. D., Weir S., Chen Y., Simpson M., Tsai S. P., Dennis M. S., Lu Y., Meng Y. G., Ng C., Yang J., Lee C. C., Duenas E., Gorrell J., Katta V., Kim A., McDorman K., Flagella K., Venook R., Ross S., Spencer S. D., Lee Wong W., Lowman H. B., Vandlen R., Sliwkowski M. X., Scheller R. H., Polakis P., Mallet W. (2008). Nat. Biotechnol..

[cit7] Hermanson G., Bioconjugate Techniques, Academic Press, San Diego, 1st edn, 1996.

[cit8] Seim K. L., Obermeyer A. C., Francis M. B. (2011). J. Am. Chem. Soc..

[cit9] Obermeyer A. C., Jarman J. B., Francis M. B. (2014). J. Am. Chem. Soc..

[cit10] Witus L. S., Moore T., Thuronyi B. W., Esser-Kahn A. P., Scheck R. A., Iavarone A. T., Francis M. B. (2010). J. Am. Chem. Soc..

[cit11] Scheck R. A., Francis M. B. (2007). ACS Chem. Biol..

[cit12] Witus L. S., Netirojjanakul C., Palla K. S., Muehl E. M., Weng C.-H., Iavarone A. T., Francis M. B. (2013). J. Am. Chem. Soc..

[cit13] Dawson P., Muir T., Clark-Lewis I., Kent S. (1994). Science.

[cit14] Sletten E. M., Bertozzi C. R. (2008). Org. Lett..

[cit15] Behrens C. R., Hooker J. M., Obermeyer A. C., Romanini D. W., Katz E. M., Francis M. B. (2011). J. Am. Chem. Soc..

[cit16] Obermeyer A. C., Jarman J. B., Netirojjanakul C., El Muslemany K., Francis M. B. (2014). Angew. Chem., Int. Ed..

[cit17] Gautier A., Juillerat A., Heinis C., Corrêa I. R., Kindermann M., Beaufils F., Johnsson K. (2008). Chem. Biol..

[cit18] Uttamapinant C., White K. A., Baruah H., Thompson S., Fernández-suárez M., Puthenveetil S., Ting A. Y. (2010). Proc. Natl. Acad. Sci. U. S. A..

[cit19] Rosen C. B., Kodal A. L. B., Nielsen J. S., Schaffert D. H., Scavenius C., Okholm A. H., Voigt N. V., Enghild J. J., Kjems J. R., Tørring T., Gothelf K. V. (2014). Nat. Chem..

[cit20] Nguyen T., Joshi N. S., Francis M. B. (2006). Bioconjugate Chem..

[cit21] Chung J. A., Wollack J. W., Hovlid M. L., Okesli A., Chen Y., Mueller J. D., Distefano M. D., Taton T. A. (2009). Anal. Biochem..

[cit22] Wakankar A., Chen Y., Gokarn Y., Jacobson F. S. (2011). MAbs.

[cit23] Hamblett K. J., Senter P. D., Chace D. F., Sun M. M. C., Lenox J., Cerveny C. G., Kissler K. M., Bernhardt S. X., Kopcha A. K., Zabinski R. F., Meyer D. L., Francisco J. A. (2004). Clin. Cancer Res..

[cit24] Le L. N., Moore J. M. R., Ouyang J., Chen X., Nguyen M. D. H., Galush W. J. (2012). Anal. Chem..

[cit25] Amandine B., Wagner-rousset E., Colas O., Ayoub D., Corva N., Dorsselaer A. V., Beck A., Cianfe S. (2014). Anal. Chem..

[cit26] Verhelst S. H. L., Fonović M., Bogyo M. (2007). Angew. Chem., Int. Ed..

[cit27] Vretblad P. (1974). FEBS Lett..

[cit28] Punna S., Kaltgrad E., Finn M. G. (2005). Bioconjugate Chem..

[cit29] Stephanopoulos N., Carrico Z. M., Francis M. B. (2009). Angew. Chem., Int. Ed..

[cit30] Christendat D., Saridakis V., Kim Y., Kumar P. A., Xu X., Semesi A., Joachimiak A., Arrowsmith C. H., Edwards A. M. (2002). Protein Sci..

