# Supplementary material for: Controlled levels of protein modification through a chromatography-mediated bioconjugation
Source: Chem Sci. 2015 Feb 27;6(4):2596–601. doi: 10.1039/c4sc03790a (PMC5495134; doi:10.1039/c4sc03790a)
Supplement: Supplementary file 1 [file SC-006-C4SC03790A-s001.pdf]

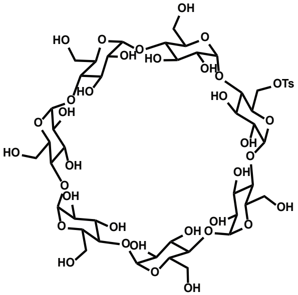

OH
HO
O
O
OH
HO
OH
O
OTs
HO
O
OH
O
OH
OH
HO
O
HO
OH
OH
O
OH
HO
OH
O
O
OH
OH
HO
OH

S1

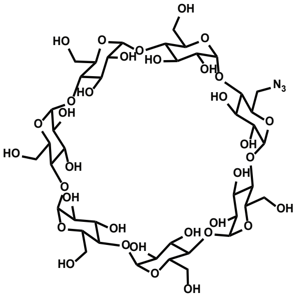

S2

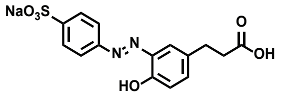

S3

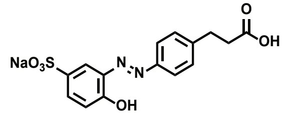

1

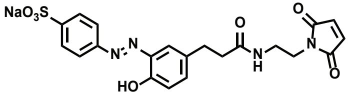

S4

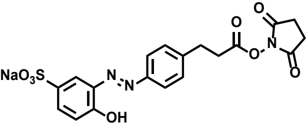

2

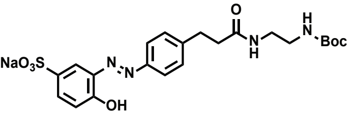

S5

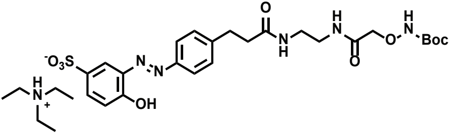

S6

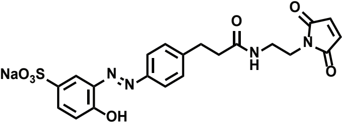

3

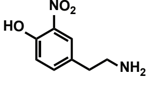

S7

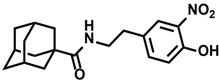

S8

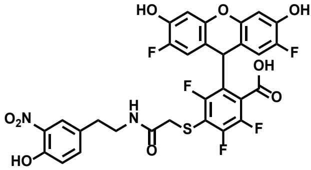

S9

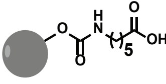

S10

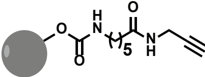

S11

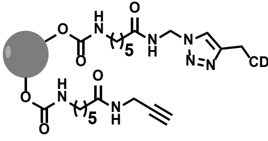

4

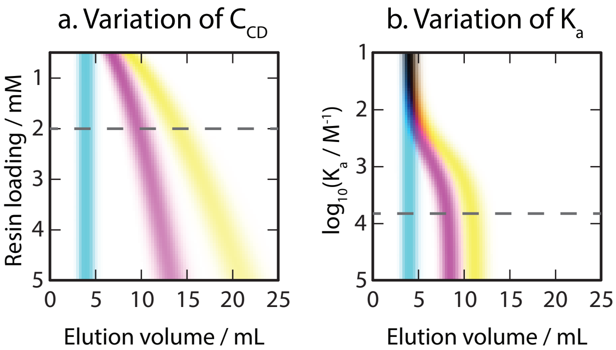

a. Variation of C_{CD}
Resin loading / mM
1
2
3
4
5
0
5
10
15
20
25
Elution volume / mL
b. Variation of K_a
$\log_{10}(K_a / M^{-1})$
1
2
3
4
5
0
5
10
15
20
25
Elution volume / mL

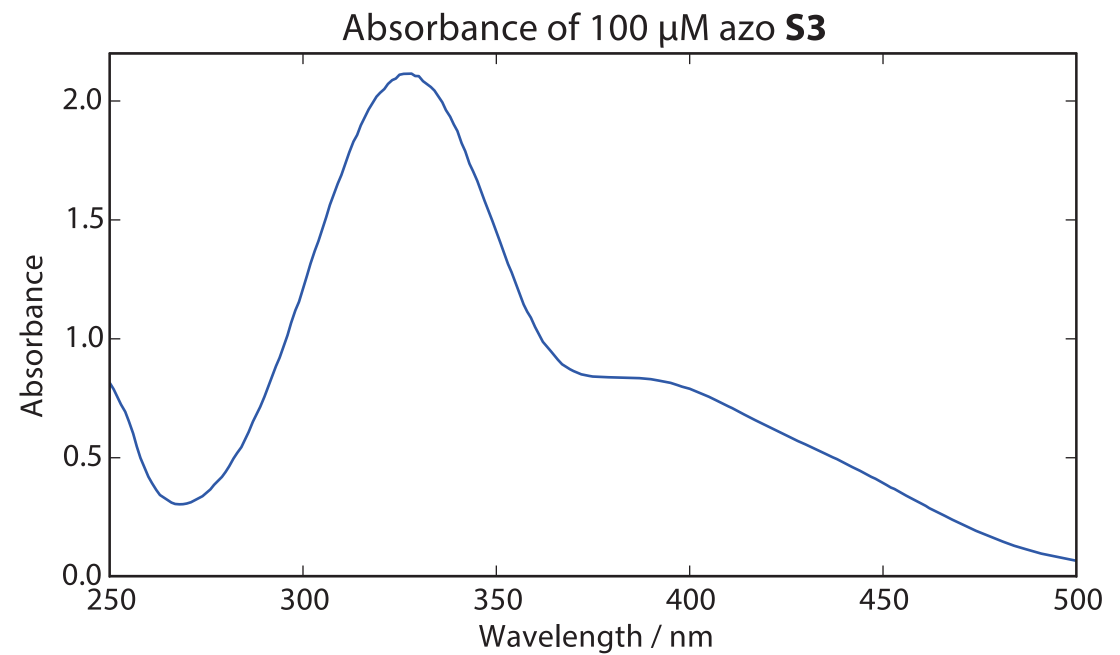
Absorbance of 100 μM azo **S3**
Absorbance
2.0
1.5
1.0
0.5
0.0
250
300
350
400
450
500
Wavelength / nm

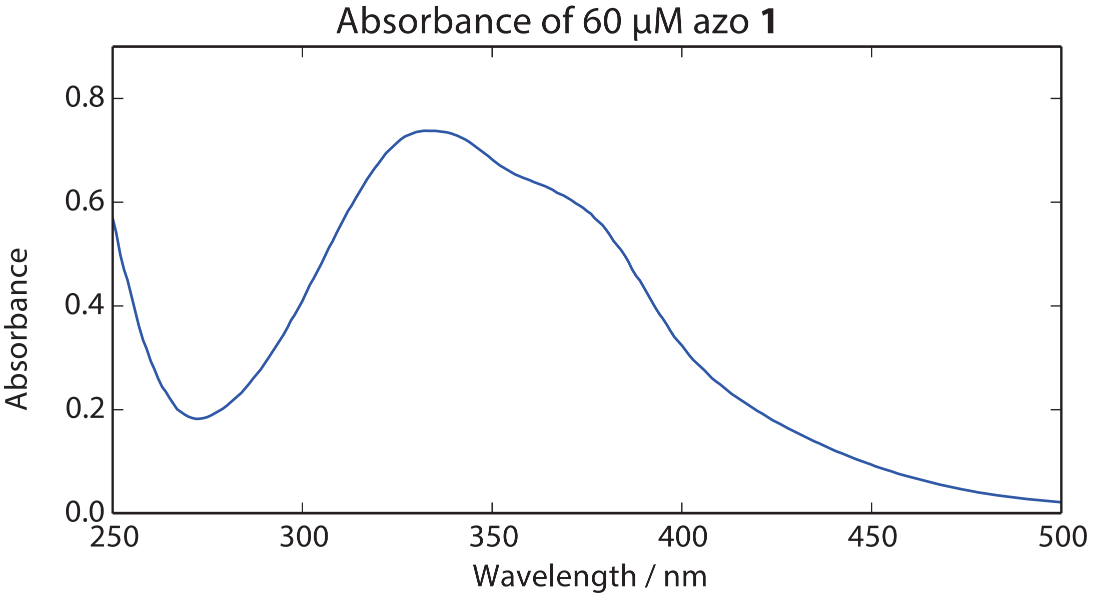

Absorbance of 60 µM azo **1**
Absorbance
0.0
0.2
0.4
0.6
0.8
250
300
350
400
450
500
Wavelength / nm

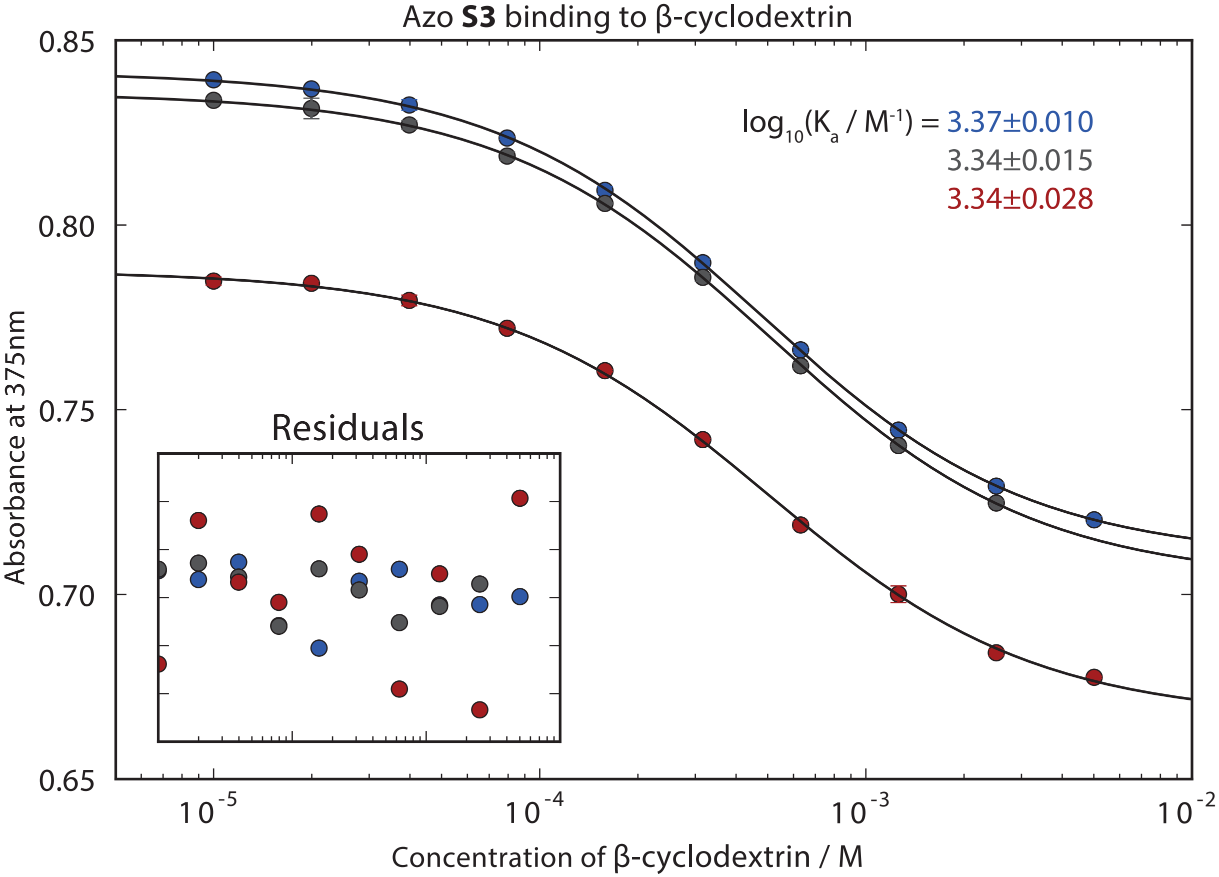

Azo **S3** binding to β-cyclodextrin
$\log_{10}(K_a / M^{-1})$ = 3.37±0.010
3.34±0.015
3.34±0.028
Residuals
0.85
0.80
0.75
0.70
0.65
Absorbance at 375nm
10^{-5}
10^{-4}
10^{-3}
10^{-2}
Concentration of β-cyclodextrin / M

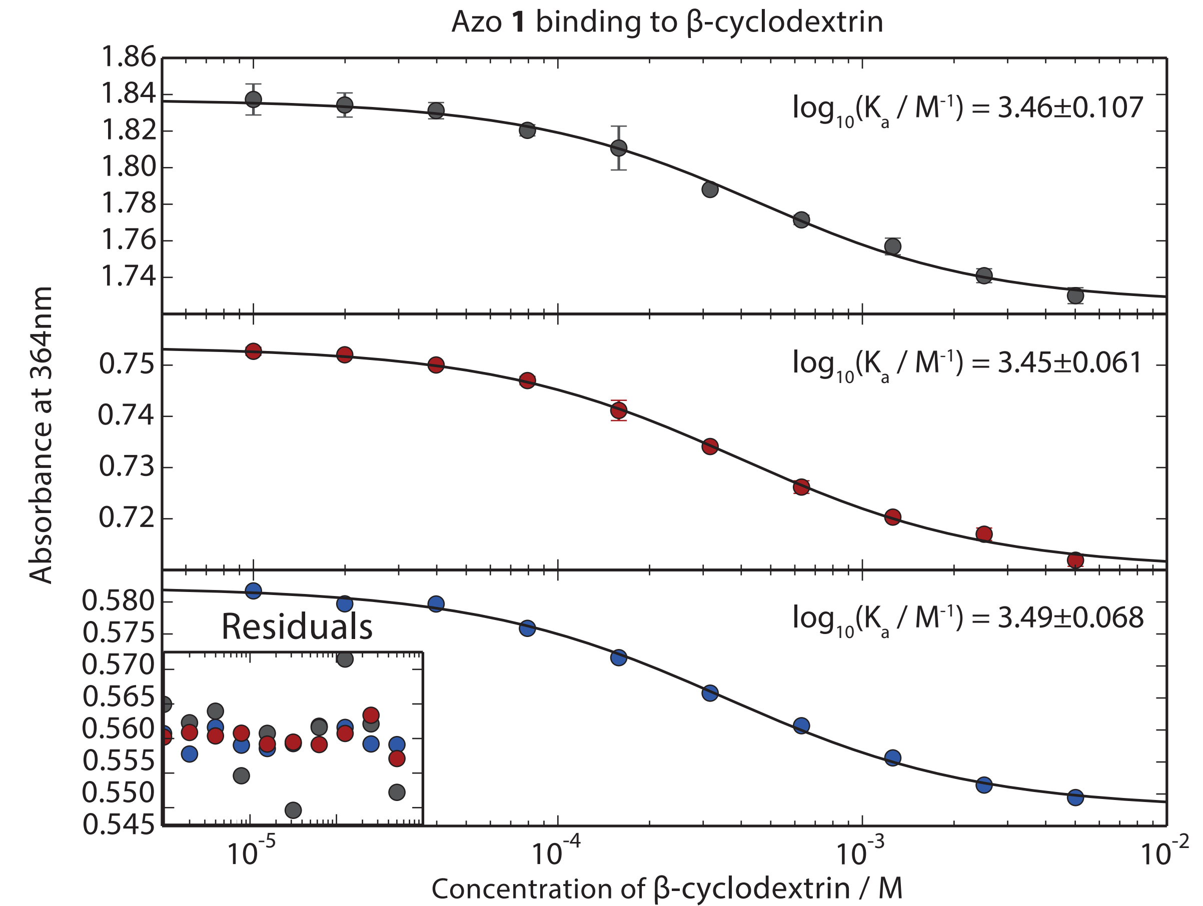

Azo 1 binding to β-cyclodextrin
log10(Ka / M-1) = 3.46±0.107
log10(Ka / M-1) = 3.45±0.061
log10(Ka / M-1) = 3.49±0.068
Residuals
Absorbance at 364nm
Concentration of β-cyclodextrin / M
1.86
1.84
1.82
1.80
1.78
1.76
1.74
0.75
0.74
0.73
0.72
0.580
0.575
0.570
0.565
0.560
0.555
0.550
0.545
10-5
10-4
10-3
10-2

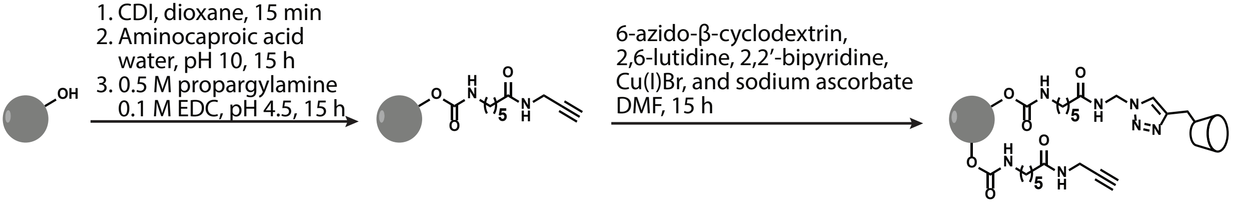

Alkyne-terminated resin

β-cyclodextrin-terminated resin

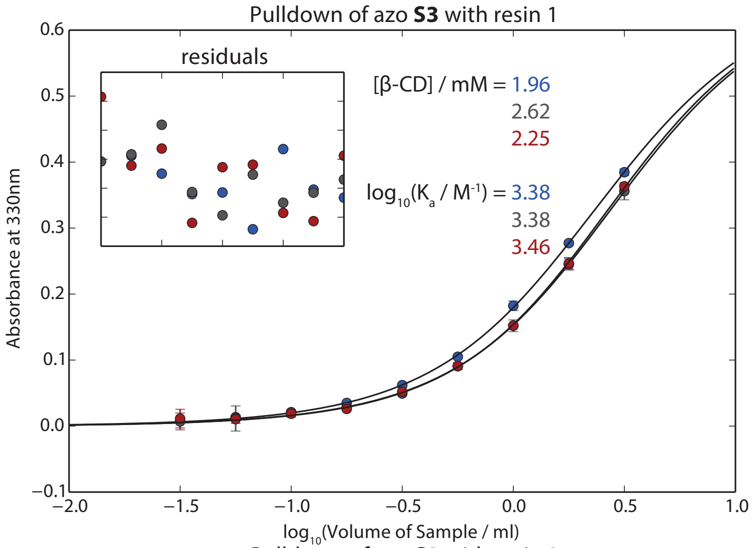
Pulldown of azo S3 with resin 1
residuals
[β-CD] / mM = 1.96
2.62
2.25
log10(Ka / M-1) = 3.38
3.38
3.46
Absorbance at 330nm
log10(Volume of Sample / ml)

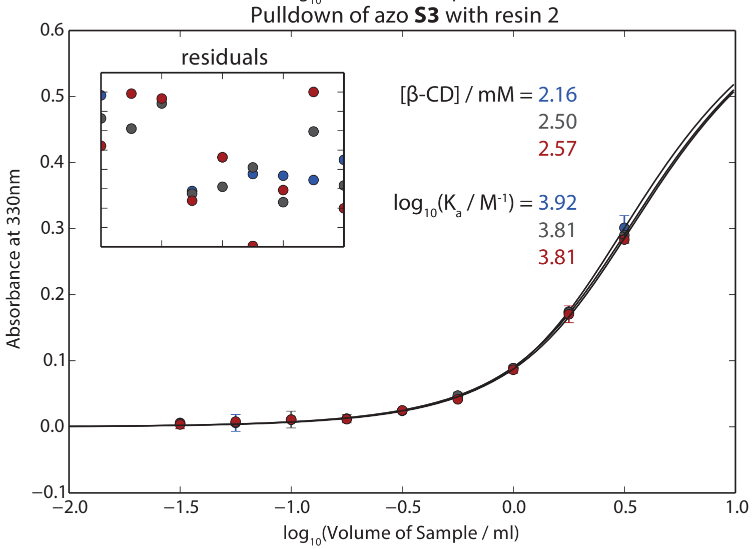
Pulldown of azo S3 with resin 2
residuals
[β-CD] / mM = 2.16
2.50
2.57
log10(Ka / M-1) = 3.92
3.81
3.81
Absorbance at 330nm
log10(Volume of Sample / ml)

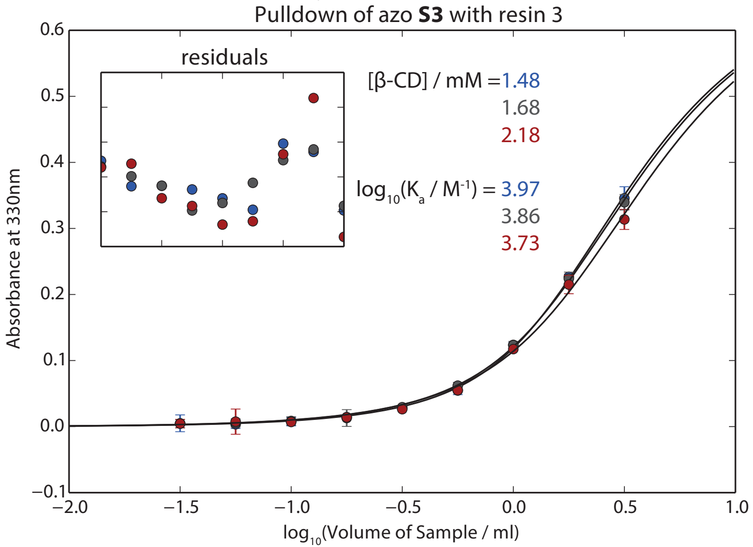
Pulldown of azo S3 with resin 3
residuals
[β-CD] / mM = 1.48
1.68
2.18
log10(Ka / M-1) = 3.97
3.86
3.73
Absorbance at 330nm
log10(Volume of Sample / ml)

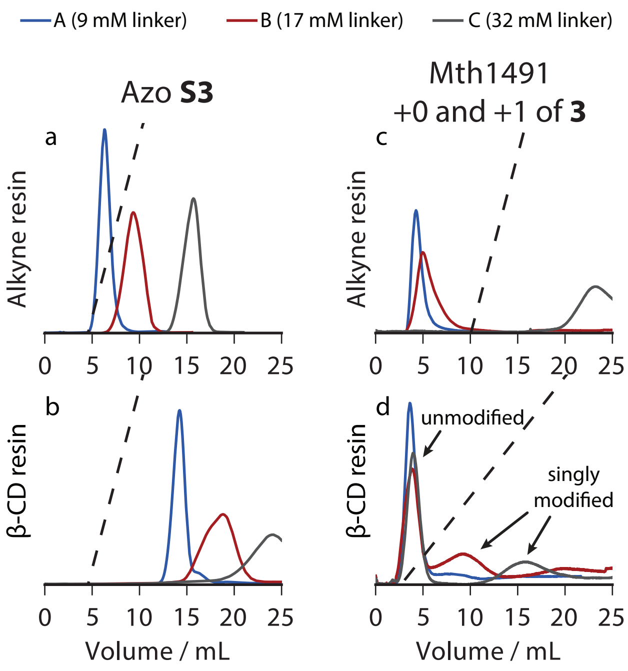
A (9 mM linker)
B (17 mM linker)
C (32 mM linker)
Azo **S3**
Mth1491
+0 and +1 of **3**
a
b
c
d
Alkyne resin
β-CD resin
unmodified
singly
modified
0 5 10 15 20 25
Volume / mL

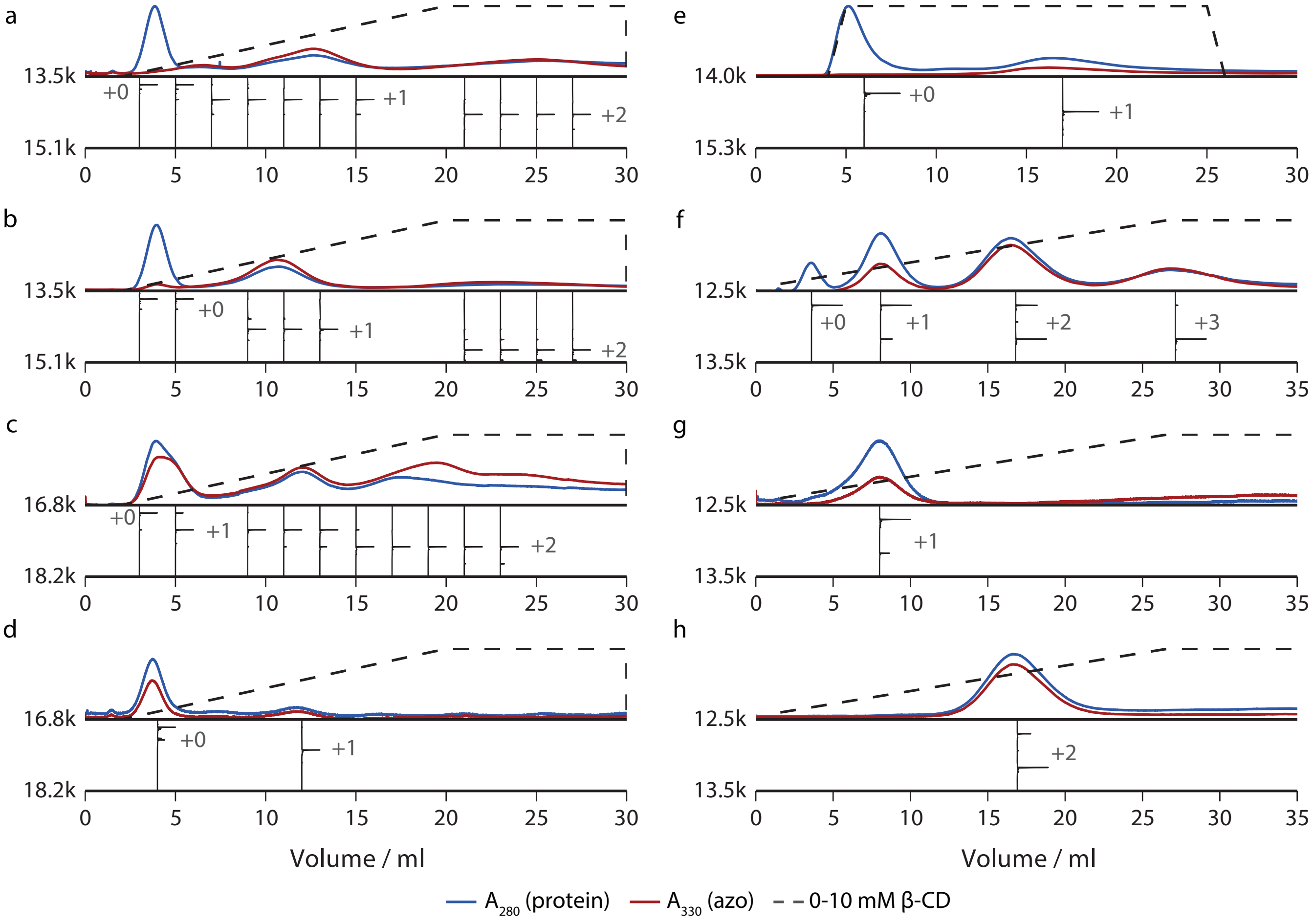
a
b
c
d
e
f
g
h
+0
+1
+2
+3
13.5k
15.1k
16.8k
18.2k
14.0k
15.3k
12.5k
Volume / ml
A280 (protein)
A330 (azo)
0-10 mM β-CD

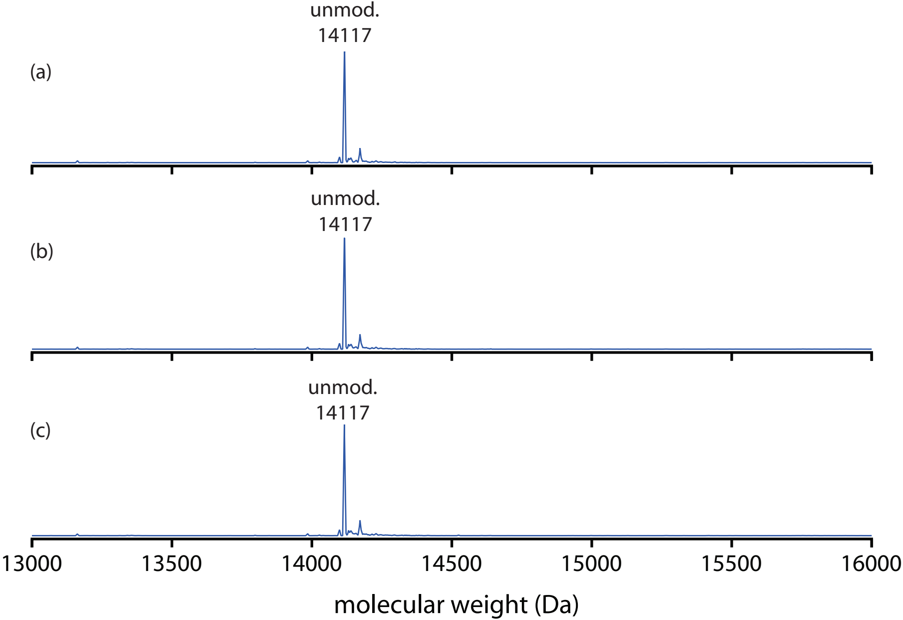
unmod.
14117
(a)
unmod.
14117
(b)
unmod.
14117
(c)
13000
13500
14000
14500
15000
15500
16000
molecular weight (Da)

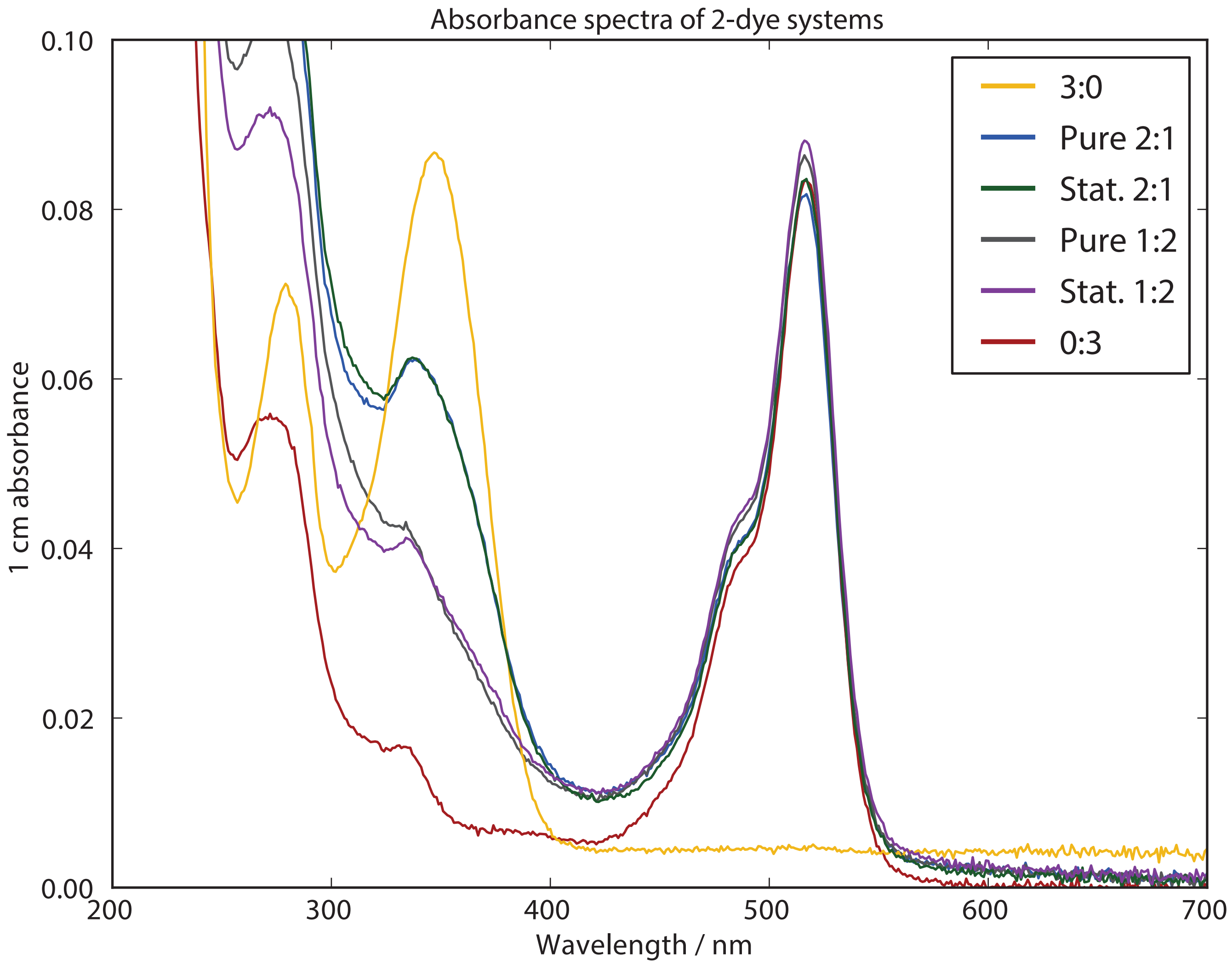

Absorbance spectra of 2-dye systems
1 cm absorbance
0.00
0.02
0.04
0.06
0.08
0.10
200
300
400
500
600
700
Wavelength / nm
3:0
Pure 2:1
Stat. 2:1
Pure 1:2
Stat. 1:2
0:3
